# Evaluation of a Pilot School-Based Physical Activity Clustered Randomised Controlled Trial—Active Schools: Skelmersdale

**DOI:** 10.3390/ijerph15051011

**Published:** 2018-05-17

**Authors:** Sarah L. Taylor, Robert J. Noonan, Zoe R. Knowles, Michael B. Owen, Bronagh McGrane, Whitney B. Curry, Stuart J. Fairclough

**Affiliations:** 1 Physical Activity and Health Research Group, Department of Sport and Physical Activity, Edge Hill University, St. Helens Road, Ormskirk, Lancs L39 4QP, UK; Robert.Noonan@edgehill.ac.uk (R.J.N.); Michael.Owen@edgehill.ac.uk (M.B.O.); Stuart.Fairclough@edgehill.ac.uk (S.J.F.); 2 Physical Activity Exchange, Research Institute for Sport and Exercise Sciences, Liverpool John Moores University, Liverpool L3 2AT, UK; Z.R.Knowles@ljmu.ac.uk; 3 School of Arts Education & Movement, Dublin City University Institute of Education, St. Patrick’s Campus, Dublin, Ireland; bronagh.mcgrane@dcu.ie; 4 Wellbeing and Public Health, Cornwall Council, Truro TR1 3AY, UK; whitney.curry@cornwall.gov.uk; 5 Department of Physical Education and Sports Science, University of Limerick, Limerick, Ireland

**Keywords:** physical activity, intervention, schools, children, accelerometry

## Abstract

Schools are key environments in which physical activity (PA) can be promoted. Various strategies and opportunities should be used to engage children in PA within schools. The aim of this study was to evaluate the effectiveness of the multi-component Active Schools: Skelmersdale (AS:Sk) pilot intervention on children’s PA and sedentary time (ST). The AS:Sk intervention was implemented for eight weeks in four schools with three control schools continuing normal practice. It consisted of eight components: active breaks, bounce at the bell, ‘Born To Move’ videos, Daily Mile or 100 Mile Club, playground activity challenge cards, physical education teacher training, newsletters, and activity homework. Child-level measures were collected at baseline and follow-up, including objectively measured PA. After accounting for confounding variables, the intervention had a significant effect on school day ST which was significantly less for the intervention children by 9 min per day compared to the control group. The AS:Sk pilot intervention was effective in reducing school day ST but significant changes in PA were negligible. To increase the efficacy of the current and future school-based interventions, authors should focus on implementation and process evaluations to better understand how schools are implementing intervention components.

## 1. Introduction

Children and young people engage in low levels of moderate to vigorous physical activity (MVPA) [1]. Worldwide data revealed 80% of 13–15 year olds do not meet the 60 min of MVPA per day guidelines [2]. Participation in physical activity (PA) during childhood years has a favourable relationship with adiposity, cardiometabolic biomarkers such as cholesterol and blood pressure, physical fitness, and bone health [3]. Psychological outcomes such as self-worth and self-esteem are also positively affected by participation in PA [4,5]. MVPA in particular is most important for health as relationships between health outcomes are most consistent and robust for PA of this higher intensity [3]. Moreover, in addition to low levels of activity, children’s sedentary time (ST) increases during the transition from primary/middle to secondary/high school [6]. Engagement in sedentary behaviours is detrimental to many aspects of health such as body composition, cardiorespiratory fitness (CRF), metabolic syndrome, and cardiovascular disease risk factors [7].

Many barriers can prevent children and young people from engaging in regular PA [8]. As a result, it has been suggested that schools are key environments for PA promotion regardless of the individual circumstances of a child [9]. Recent government recommendations state that half (at least 30 min) of the daily recommendation for MVPA should be accrued during school hours [10,11]. Recommendations for sedentary time are less prescriptive and specific, although efforts to reduce sedentary behaviours and minimise extended periods spent sedentary across the whole day and within schools are advocated [11,12,13,14].

Within comprehensive school PA programmes (CSPAP) [15] the use of a variety of strategies and opportunities is advocated to promote PA within schools, for example during the school day, before and after school, within physical education (PE), and with involvement from staff and family/community [15]. Results from a 2015 meta-analysis indicated that as the number of CSPAP components included in an intervention increased, the effect size associated with change in daily PA also increased [16].

A comprehensive intervention perspective with a focus on multiple-level factors exemplifies a socio-ecological approach [17]. Action Schools! BC (AS! BC) is an ongoing example of an intervention underpinned by the socio-ecological model [18], and which resulted in PA increasing through activities implemented across six different school components named ‘action-zones’ [19]. Literature reviews have further supported this approach to intervention design, stating that interventions targeting different levels of the socioecological model and those that are multi-component in nature can have a positive impact on PA levels [20,21,22,23].

That being said, multi-component interventions are not always successful at increasing PA [24,25]. Multi-component interventions are difficult to put into practice and a lack of implementation, with schools not implementing as intended has previously been reported [24]. More recently, a more pragmatic approach to PA promotion has been proposed which includes the expansion, extension, and enhancement of PA opportunities (theory of expanded, extended, and enhanced opportunities (TEO)) [26]. The use of this approach allows researchers to target various levels within an ecological model but additionally and importantly, identify appropriate targets [26].

The Active Schools: Skelmersdale (AS:Sk) pilot multi-component clustered randomised control trial (RCT) was designed to promote PA across the school day through multiple opportunities which could be integrated into every day school life and implemented by school staff. The aim of this study was to evaluate the impact of the AS:SK intervention on children’s MVPA and ST, and health indicators.

## 2. Materials and Methods

### 2.1. Participants

This study is the third phase of the AS:Sk project (ClinicalTrials.gov registration: NCT03283904). Seven primary schools within Skelmersdale, a low-income town, within West Lancashire, UK, participated in the project [27]. Using a sample size calculation that accounted for the pre-determined number of schools, 100 participants (50 per group) were required for a clustered RCT design with seven schools. This calculation was based on AS:Sk study 1 findings and assumed 15 participants per cluster, an intracluster correlation of 0.04, an alpha level of 0.05 and power of 90% [28,29]. Following ethical approval from the University Research Ethics Committee (ref #SPA-REC-2016-342), schools received the relevant paperwork to inform each Year 5 child (*n* = 239, age 9–10 years) about the study. Passive (“opt-out”) parental consent were obtained in six of the schools, one school chose to use active parental consent, and children completed informed assent forms prior to data collection. This process resulted in 232 participating children (97% recruitment rate).

### 2.2. Study Design

**Figure 1:**
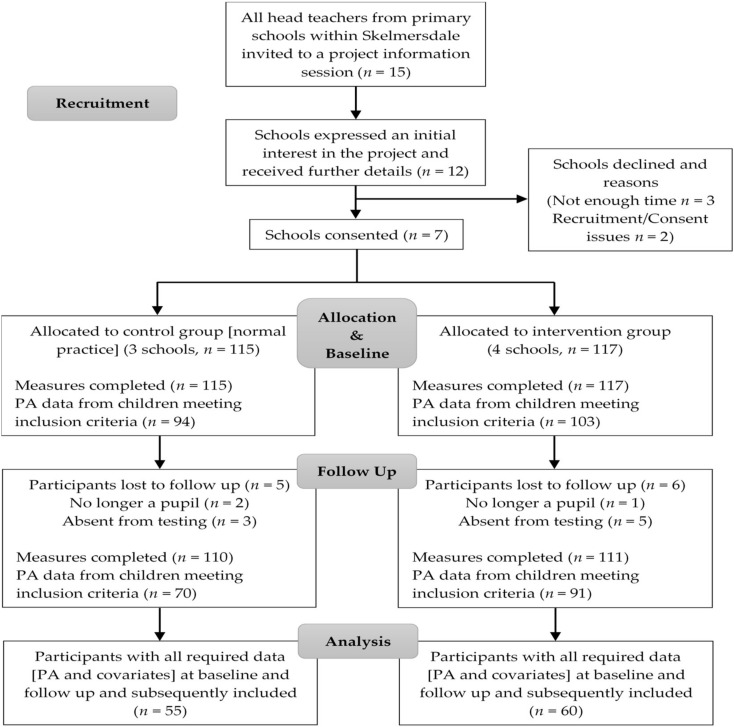
Flow of schools and participants through the study.

Following the collection of baseline measurements, schools were randomly assigned to either intervention or control groups by a member of the faculty unconnected to the study. This randomisation was not blinded due to the nature of the intervention. There was a one-week gap between the allocation of groups and the beginning of the intervention period to allow for the teachers to plan and organise intervention components into their future school plans. Control schools were informed via email of their selection and agreed to continue with their usual timetabled amount of playground breaks and PE lessons without any additional time allocated for PA participation. Details of the flow of participants through the study from baseline to follow up are shown in Figure 1.

### 2.3. Intervention

The Consolidated Standards of Reporting Trials (CONSORT) guidelines extension for clustered RCT were followed for reporting the results of the AS:Sk intervention [30]. The intervention duration was eight weeks and it consisted of eight components. These were active breaks (ABs), bounce at the bell, ‘Born To Move’ (BTM) videos, Daily Mile (DM) or 100 Mile Club (MC), playground activity challenge cards, PE teacher training, newsletters, and activity homework. All intervention approaches were designed to have no financial cost to the project or schools to implement. A description of each intervention component with the recommended implementation duration and frequency per school day or week was presented to each participating class teacher who was asked to adhere to this guidance. These details are presented in Table 1. Schools were given the freedom to implement the components during the school day when it best suited their own timetable, whilst adhering to the duration and frequency guidelines. The consultation of relevant school-based intervention literature and findings from phase two of the AS:Sk project which piloted three components (ABs, BTM videos, recess intervention; unpublished data), informed selection of the current components. The components aligned with elements of the socio-ecological model [17], the youth physical activity promotion model (YPAPM) [31], and TEO [26].

### 2.4. Measures

The primary outcome for this study was school day MVPA. The secondary outcomes were achieving 30 min MVPA during the school day, school day ST, whole weekday ST and PA levels, CRF, and body size (BMI z-score). Measurement protocols at baseline and follow up were the same at both time points and took place within the school grounds. Baseline measures were taken in September 2017, with follow up measures taken in November and December 2017.

#### 2.4.1. Physical Activity

Children wore an ActiGraph GT9X triaxial accelerometer (ActiGraph, Pensacola, FL, USA) which were each initialised to record raw accelerations at a frequency of 100 Hz. Children were instructed to wear the accelerometer for seven days at all times (24 h·day^−1^), except when engaging in water-based activities such as bathing and swimming. Data was downloaded using ActiLife version 6.11.9 (ActiGraph) and saved in raw format as GT3X files. Raw data files were processed in R (http://cran.r-project.org) using GGIR which converted the raw triaxial accelerometer signals into one omnidirectional measure of acceleration termed the Euclidean norm minus one (ENMO; vector magnitude taken from the three axes minus the value of gravity with negative values rounded up to zero) [45,46]. ENMO values were averaged per 1 s epoch over each of the seven monitored days [47]. Accelerometer non-wear was determined using the method of van Hees et al. [45], which has been applied previously in studies involving children [47,48,49]. Published ENMO prediction equations were used to identify cut-points for classifying activity as MVPA (3 metabolic equivalents (METs; child-specific); 201 mg) [50]. As there is no consensus as to the most appropriate ENMO ST cut-points [51], we also applied the Hildebrand et al. [50] regression equations using 1.5 METs, which resulted in values of 50 mg. Minimum wear time to be included in the analysis was set to 10 h for a minimum of three weekdays at both baseline and follow up [52]. The time periods explored in the analyses included the school day (defined by schools as between the time the timetable begins and the time children are dismissed) and also whole week day (defined as 7 am to 10 pm).

#### 2.4.2. Anthropometrics

Stature was assessed to the nearest 0.1 cm using a portable stadiometer (Leicester Height Measure, Seca, Birmingham, UK). Body mass was assessed to the nearest 0.1 kg (813 scales, Seca). Body weight in kilograms divided by height in meters squared gave the body mass index (BMI) of each participant. BMI z-scores were assigned [53] and age and sex specific BMI cut-points established children as normal weight or overweight/obese (those who were underweight were grouped into the normal weight category) [54]. Waist circumference was measured to the nearest 0.1 cm using an anthropometric tape measure, and the percentage of waist circumference-to-height ratio (%WHtR) was calculated as a measure of central adiposity [55]. Gender-specific equations were used to predict children′s age from peak height velocity (APHV), as a proxy measure of biological maturation [56].

#### 2.4.3. Cardiorespiratory Fitness (CRF)

The 20 m multistage shuttle run test was conducted to provide an estimate of CRF [57]. The total number of shuttles completed by each participant was recorded as a proxy measure of CRF. This test has been previously used with children of a similar age to those in the current study [43,58].

#### 2.4.4. Psychological Constructs

A paper questionnaire pack was administered which included eight items measuring PA self-efficacy [59] and 16 items measuring PA enjoyment [60]. All items were scored using a 5-point scale ranging from 1 (“Strongly disagree”) to 5 (“Strongly agree”). These questionnaires have previously demonstrated strong factorial validity [59,60].

#### 2.4.5. Socioeconomic Status (SES)

Neighbourhood-level socioeconomic status (SES) was calculated using the 2015 Indices of Multiple Deprivation (IMD) [61]. The IMD is a UK government-produced deprivation measure for England comprising income, employment, health, education, housing, environment, and crime. IMD rank scores were generated from parent-reported home post codes using the National Statistics Postcode Directory database. Every neighbourhood in England is ranked from one (most deprived area) to 32,844 (least deprived area).

### 2.5. Statistical Analysis

Descriptive statistics (mean and standard deviations) were calculated for the outcomes of all participants at baseline and follow-up. Multilevel modelling was performed using MLwiN Version 2.36 (Centre for Multilevel Modelling, University of Bristol, UK) [62] to determine the effects of the intervention. Multilevel modelling was appropriate for use in this study given the design of children clustered within the seven participating schools. Therefore, a 2-level data structure was used with children defined as the first level of analysis, and schools as the second level of analysis.

Continuous outcome variables were school day ST, light PA (LPA) and MVPA, whole weekday ST, LPA and MVPA, CRF and BMI z-score. The dichotomous outcome variable studied (thus logistic multilevel analysis) was achieving 30 min MVPA/school day. Regression coefficients for the group variables (‘0’ indicating control schools and ‘1’ indicating intervention schools) reflected between-group differences in the outcome measures (adjusted for baseline values and covariates). Initially, ‘crude’ interaction analyses were conducted with only the grouping variables and the outcome variable at baseline included in the model [63]. Potential confounding covariates were then added to ‘adjusted’ models whilst still controlling for baseline outcome variables. These potential confounding covariates were selected based on previous research which has deemed them to be influential to the outcomes and depending on the outcome, included gender [1,64], SES [65,66], body size [67,68], CRF [69,70], PA self-efficacy [31], PA enjoyment [31], accelerometer wear time, and whole weekday ST and MVPA [68,71,72]. Regression coefficients from the models were assessed for significance using the Wald statistic and the following equation, (regression coefficient/standard error)^2^. Statistical significance was set at *p* < 0.05.

The evaluation of potential effect modification was also carried out on several dichotomous covariates (gender, weight status, central obesity risk, and fitness status). These analyses determined whether the intervention effects were different for the subgroups. Interaction terms were added to the models, consisting of a multiplication of the main determinant (intervention) and the potential effect modifier [63]. Due to the reduced power which interaction terms have, statistical significance for this analysis was set at *p* < 0.1 [63].

## 3. Results

### 3.1. Preliminary Results

Descriptive statistics are displayed in Table 2 for all participants and by gender, for baseline and follow up measures.

### 3.2. Intervention Effects

Table 3 shows the intervention effects on each outcome. In the adjusted models, time spent engaged in ST during the school day was significantly less for the intervention children compared to the control group (−9.0 min; *p* = 0.01). There were no intervention effects on any of the remaining outcome measures, although the trends for school day PA and CRF were in a favourable direction. The odds of achieving 30 min of MVPA per school day was 2.79 times higher in the intervention group compared to the control group, however this did not reach significance (*p* = 0.07).

### 3.3. Sub-Group Analyses

There were no post-intervention interaction effects in any of the dichotomous variables (sex, weight status, central obesity risk, fitness status) on the outcomes of school day ST and PA, whole day ST and PA, BMI z-score, and CRF.

## 4. Discussion

This study aimed to (1) assess the impact of the AS:Sk multi-component intervention on the primary outcome of school day MVPA, and (2) assess the impact of the AS:Sk multi-component intervention on the secondary outcomes of achieving 30 min MVPA/school day, school day ST, whole weekday ST and PA levels, CRF and body size. Overall, after accounting for confounding variables, the intervention had a significant effect on school day ST which was significantly less for the intervention children by 9 min per day compared to the control group. Trends were observed for favourable changes in school day LPA, PA, MVPA, achieving 30 min school day MVPA, and CRF, however these did not reach significance.

The AS:Sk intervention demonstrates school-based PA components which are novel in their ability to target various time points in the school day with no financial costs to the school. The significant effects that the intervention had on ST are consistent with previous research. For example, the Finnish Schools on the Move study, which allowed schools to plan their own interventions with strategies such as longer recess periods, increased use of equipment during the school day, and staff training, reported decreased ST at 1.5 year follow-up in children similar in age to those in AS:Sk [73]. In contrast, the Active Living multi-component school-based intervention, which used techniques to target PA in school, before, and after school with active transport, and also during leisure time observed a general increase in ST at 12 months follow-up (2.2% more daily time spent in sedentary behaviour), which the authors speculated could have been due to the participants increase in age [25]. Given the short follow up period in the current study, it is difficult to establish whether the initial positive impact on ST would be sustained long term, inhibiting the anticipated age-related increase. Project timescale and subsequent funding precluded the utilisation of a longer-term intervention period and follow up evaluations.

A significant intervention effect on school day ST has implications for both public health policy and child health outcomes. Public health guidelines in both the UK and other countries recommend that overall sedentary time should be limited in children and young people [12,13,14]. Moreover, research has explored the relationship between ST and health indicators, subsequently highlighting the detrimental effects that ST can have on child health. For example, time spent being sedentary is positively associated with BMI z-score, and negatively associated with fitness in children and youth (aged 6–17 years) [74].

Results indicated a modest and non-significant increase in school day MVPA of 1.5 min. Sutherland and colleagues also reported modest increases in MVPA after the implementation of their multi-component school-based programme, ‘PA 4 Everyone’ [41]. Differences to control students were significant, with 3.9 more minutes of MVPA per day accumulated by intervention students [41]. Conversely, the ‘Active Living’ multicomponent school-based PA intervention had no significant effect on MVPA per day and saw a general reduction in PA [25].

The addition of even small amounts of MVPA to the school day may be beneficial to physical health, particularly when compared to interventions which see negative outcomes and also when the age-related decline in MVPA is considered [75]. However, the meaningfulness of potential benefits could be questioned. The addition of MVPA does predict positive effects with decreased adiposity, whilst the replacement of MVPA with any other movement behaviour predicts negative effects with higher adiposity and lower CRF [76,77]. However, these results are based on 15 min reallocations of time which is considerably more than the intervention effect on MVPA in the current study. Researchers and practitioners should focus on developing sustainable strategies for increasing MVPA participation during the school day given its significant importance for physical health. Understanding how interventions are implemented within schools from the perspective of teachers and students alike, may help in the development of successful school-based techniques. The process evaluation of interventions is advocated by the UK Medical Research Council (MRC) and can play a crucial role in understanding and learning from findings [78,79]. Despite this, implementation data are rarely reported in the literature and a lack of standardised definitions and measurements of implementation contributes to this [79]. A review into the barriers and facilitators to the implementation of PA policies in schools concluded that the body of literature surrounding this topic area from a theoretical perspective was scarce [80]. Implementation of PA in the classroom setting has received more coverage in the literature recently, including perspectives from teachers which has provided useful and important considerations for future interventions [81,82].

There were no significant intervention effects on whole weekday movement behaviours (including out of school hours). A previous systematic review concluded that school-based interventions had no effect on leisure time PA [83]. Whilst results were not significant, intervention effects on whole weekday PA were in the negative direction. This could suggest that children compensated for the increased PA opportunities they were provided with during the school day by decreasing their leisure time PA. This theory has also been suggested by previous interventions in which increases in school day MVPA did not translate into positive effects across the day [73]. An intervention which increased the number of compulsory PE lessons found that the percentage of time spent in MVPA during school was greater; however, the percentage of time spent in MVPA out of school was lower when both time periods were compared to normal schools [84]. Further PA compensation research has also suggested that for every additional 10 min spent in MVPA, children engaged in 5 min less the following day [85]. That being said, not all interventions report compensation effects, for example a review of school-based interventions found five in total which were effective at increasing overall PA [86]. AS! BC is one of these interventions that was effective at increasing overall PA [87]. Activities implemented across six action zones in this intervention included extracurricular and family and community, these zones in particular may have been the important factor which limited PA compensation outside of the school day [87].

The CSPAP approach to PA promotion comprises of five different components or points of intervention which includes PA before and after school [15]. Whilst attempts were made to target the out of school period with the PA homework component of the AS:Sk intervention it would appear that more substantial efforts are needed, for example with school-based extracurricular PA opportunities, rather than PA that requires children to engage with in the home environment. Many barriers to participation in out of school PA exist, including parental reported barriers such as safety concerns [88]. Screen time has also been reported by parents as a barrier, particularly as it is seen as the ‘norm’ for children to engage and therefore parents struggle to limit it [88,89]. Parents have reported that engagement in family-based PA intervention programmes would be the most effective way to increase their child’s PA [88]. The out of school time period for PA participation requires more attention, even from interventions which are primarily designed as school-based, in which the out of school barriers to PA participation and the desired family-based sessions should be considered.

The AS:Sk intervention had several strengths. Firstly, it was developed through prior formative research and was theoretically underpinned by conceptual behaviour change models [17,26,31]. This approach adheres to MRC guidelines for the development of complex interventions [90]. In addition, school staff were provided with the flexibility to implement the PA components when it best suited their class or school. This approach is most feasible in the “real-world” school setting in which unpredictable changes to timetables can happen, thus programme flexibility has previously been reported by teachers as a facilitator to implementation [19]. There was also no financial cost to the schools or the project. This would suggest that the intervention can be self-sustained by schools alone and, therefore, has potential for long-term implementation, although the teacher burden relating to planning and implementation should not be understated. The use of objectively measured PA to assess the intervention effect is an important strength of the study. Furthermore, the use of raw accelerations avoids the uncertainty of pre-processed data such as counts and the possibility that signal-filtering methods alter study results [91,92]. A limitation of the study is the modest sample size, which may have resulted in a lack of power in the statistical test outcomes, particularly the positive outcomes which did not reach statistical significance. The number of children who met the accelerometer wear-time criteria at both baseline and follow up measures also impacted on the final sample size. A further limitation was the timing of the follow up measures in both control and intervention schools. By necessity, measures were taken at an atypical school period, in the final few weeks before Christmas. It is in this period that school timetables are often disregarded and festive activities sometimes replace usual practice. Thus, the activity of children may not be representative of the rest of the school year. Intervention schools in particular may not have implemented the intervention in these final school weeks as they may have done so earlier in the school term. Furthermore, given that intervention implementation was sustained by school staff only, without any external support, it is likely that there were differences in implementation between participating schools. Gaining an accurate and objective record of implementation frequency across the eight-week period within each participating school may require daily researcher visits during the intervention period, which was not possible due to the time constraints of the research staff. Alternatively, teacher logs could be used, but these may be more subject to bias. Quantitative data to illustrate implementation frequency across the eight-week period was, therefore, not available, and it is acknowledged that differences in implementation frequency between schools likely impacted the results. The lack of a more long-term follow up measurement period was also a limitation. Given that follow up measurements were taken only eight weeks after implementation it is difficult to understand the sustainability of the intervention. The overall short intervention implementation period of eight weeks is also a weakness of the study, as interventions of longer duration have been shown to be more effective [86].

## 5. Conclusions

The AS:Sk multi-component school-based PA intervention had a significant positive effect on school day ST. There were no significant intervention effects on any of the other outcome measures. The small sample size of the current study was an important limitation within the study and may have contributed to the analyses lacking power. The school day period should continue to be a priority. Its importance for PA participation has previously been highlighted, and this study indicates that positive effects on ST in particular are achievable across the school day. Modifications to out-of-school components would be beneficial to avoid any compensation effects on PA participation. The AS:Sk intervention has potential to be scaled up to a full trial following modifications based on the results of this pilot study. Future research should focus on exploring ways in which MVPA participation can be increased during the school day. This may be with the development of appropriate school-based techniques or, conversely, focusing on how to improve the implementation of established techniques (such as the components of the current intervention) through process evaluation research.

## Figures and Tables

**Table 1:** Detail of each intervention component.

Intervention Component	Content Description	Phase 2 Findings	Associated/Supportive Research	Conceptual Model/Theory	Duration	Frequency
Active Breaks	Twenty-three activity cards were created with pictures on the front demonstrating the activity and instructions on the back. All activities were designed for use within the restricted space of a classroom. Each activity card was designed to last for 30 s. *(Delivery: class teacher)*	Deemed feasible and acceptable. No changes needed.	Pilot primary school AB study with a similar 5-min implementation protocol [32]. ABs reported to improve PA during school [33].	SE YPAPM TEO	5 min.	x1/day.
Bounce at the bell	Teachers were provided with a suggested jump routine (star jumps, tuck jumps) to perform whenever the bell sounded in class (usually for morning break, lunch break and the end of the school day). The jumps were to be performed once the lesson had finished just before leaving the classroom. *(Delivery: class teacher)*	N/A	Used in a PA school-based intervention for increasing bone strength (no PA outcomes) [34]. Reported as a simple classroom-based exercise without the need for equipment or access to a gym, requiring only 3 min of the school day [35].	SE YPAPM TEO	1–2 min.	x3/day.
Born To Move videos	Videos provided by Les Mills (free access videos available on http://www.lesmillsondemand.com), included instructor led high-intensity motor skills set to contemporary music, designed to improve health-related and skill-related fitness. Videos required hall/gym space with a projector screen connected to an internet enabled device. *(Delivery: class teacher)*	Daily implementation reduced due to hall/gym accessibility barrier.	Evaluation of BTM pilot programme concluded that live 30-min BTM lessons delivered by a trained instructor engaged children in significantly more MPA than during regular PE [36].	SE YPAPM TEO	10 min.	x2/week.
Daily Mile or 100 Mile Club	Schools planned an outdoor route around school grounds. If the route was smaller than a mile, the number of laps required to achieve the mile was calculated. For the 100 MC, each child received a recording sheet to record miles accumulated. For the DM option, no tracking of distance ran was required. *(Delivery: class teacher)*	N/A	Short-term follow up results of a study implementing 100 MC in lower-income schoolchildren indicated significant positive effect on ST [37]. The DM is cited by the UK government as an option for schools to deliver PA [10].	SE YPAPM TEO	15 min.	x1/day (DM). x3/week (100 MC).
Playground activity challenge cards	There were 5 games in total which all included 5 different activity cards. Activities were easy-to-perform exercises designed for children to follow independently without the need for any equipment (apart from a ball in one of the games) or the need for teachers to set up or assist with games. They were placed around the playground in visible places (tied to gates/faces, stuck to classroom windows). *(Delivery: child independent*/*playground staff)*	Challenges/games designed for children to follow independently due to teacher barriers cited.		SE YPAPM TEO	5 min per game.	Every recess break.
PE teacher training	The school sport coach or PE teacher in each intervention school were sent access to an online training session (immediately after intervention allocation, the week prior to the intervention period). The focus of the online content was how to increase high intensity PA and reduce time spent standing still during PE. Access to follow-up support via email was provided. *(Delivery: PE teacher)*		Supportive, Active (high levels of PA, minimal transition time), Autonomous (opportunity for student choice), Fair, Enjoyable (SAAFE) framework used to guide staff for the planning and delivery of their PE lessons [38]. LET US Play principles also highlighted to staff [39]. Including removing lines, eliminating elimination, reducing team sizes and rethinking space, equipment and rules.	SE YPAPM TEO	N/A	Every PE lesson.
Newsletters	Information relating to PA and its importance for health and wellbeing were sent to schools. Schools were asked to insert messages into their school newsletter which was sent home to all parents (most commonly online via an email or through the school website).	N/A	Use in previous school-based PA interventions as a means for engaging parents [40,41,42].	SE YPAPM		Weekly/2 weeks (school dependent).
Activity homework	Children received a homework pack which included a letter to parents and 10 different PA challenges. A separate pack of the individual challenges on small pieces of paper were also provided for children to take home if their original pack had been lost at home. Children received a weekly diary to complete whenever they had done PA at home. A blank class chart was provided to populate with names and update every week with school rewards for those who completed the most PA at home.	N/A	Use in previous school-based PA interventions [43,44].	SE YPAPM TEO		Encouraged to be x1/day.

AB, active break; PA, physical activity; SE, socio-ecological model; YPAPM, youth physical activity promotion model; TEO, theory of expanded, extended, and enhanced opportunities; BTM, born to move; DM, daily mile; 100 MC, 100 mile club; PE, physical education.

**Table 2:** Descriptive characteristics of participating children (control and intervention, baseline and follow up; mean (standard deviation) where applicable).

	Baseline					Follow Up			
Measure	Sex	*n*	Control	*n*	Intervention	*n*	Control	*n*	Intervention
Stature (cm)	Boy	54	137.5 (7.2)	60	136.9 (5.1)	52	138.6 (7.2)	56	137.7 (4.9)
	Girl	60	136.7 (6.7)	58	137.8 (6.0)	58	137.5 (6.5)	54	139.0 (6.2)
	All	114	137.1 (6.9)	118	137.3 (5.5)	110	138.0 (6.8)	110	138.3 (5.6)
Body mass (kg)	Boy	54	34.9 (8.8)	59	33.7 (6.3)	52	35.9 (8.8)	55	34.0 (6.3)
	Girl	60	35.2 (8.5)	58	37.1 (8.1)	56	35.7 (9.1)	54	38.0 (8.5)
	All	114	35.1 (8.6)	117	35.4 (7.4)	108	35.8 (8.9)	109	36.0 (7.7)
BMI (kg·m^2^)	Boy	54	18.3 (3.2)	59	17.9 (2.6)	52	18.5 (3.2)	55	17.8 (2.6)
	Girl	60	18.7 (3.5)	58	19.5 (3.6)	56	18.7 (3.7)	54	19.6 (3.6)
	All	114	18.5 (3.3)	117	18.6 (3.2)	108	18.6 (3.5)	109	18.7 (3.3)
BMI z-score	Boy	53	0.7 (1.2)	56	0.5 (1.1)	51	0.7 (1.1)	53	0.5 (1.0)
	Girl	60	0.7 (1.3)	57	0.9 (1.2)	56	0.5 (1.2)	53	0.9 (1.2)
	All	113	0.7 (1.2)	113	0.7 (1.2)	107	0.6 (1.2)	106	0.7 (1.2)
Overweight/Obese (%)	Boy	53	22.6	56	21.4	51	25.5	53	18.9
	Girl	60	35.0	57	43.9	56	35.7	53	47.1
	All	113	29.2	113	32.7	107	30.8	106	33.0
Waist circumference (cm)	Boy	54	63.7 (9.5)	59	63.5 (7.8)	52	66.7 (9.1)	55	63.8 (6.7)
	Girl	60	63.7 (9.4)	58	65.9 (8.8)	56	65.1 (10.1)	54	66.1 (8.5)
	All	114	63.7 (9.4)	117	64.7 (8.3)	108	65.9 (9.6)	109	64.9 (7.7)
Maturity offset (y)	Boy	51	−3.2 (0.3)	57	−3.3 (0.2)	51	−3.0 (0.4)	54	−3.0 (0.3)
	Girl	60	−2.2 (0.4)	57	−2.1 (0.3)	57	−1.8 (0.5)	53	−1.8 (0.5)
	All	111	−2.7 (0.7)	114	−2.7 (0.6)	108	−2.3 (0.7)	107	−2.4 (0.7)
CRF (Number of shuttles)	Boy	52	36.7 (18.3)	59	33.1 (15.2)	50	34.1 (18.9)	57	36.2 (17.6)
	Girl	58	28.2 (13.3)	55	25.1 (11.4)	57	25.3 (12.5)	54	25.2 (11.6)
	All	110	32.3 (16.3)	114	29.2 (14.0)	107	29.4 (16.3)	111	30.9 (15.9)
IMD Rank	Boy	51	5618.8 (5324.0)	59	6379.4 (7995.8)		*N*/*A*		*N*/*A*
	Girl	58	5811.1 (6396.3)	56	8322.6 (8497.7)		*N*/*A*		*N*/*A*
	All	109	5721.1 (5892.7)	115	7325.7 (8265.5)		*N*/*A*		*N*/*A*

BMI, body mass index; CRF, cardiorespiratory fitness; IMD, indices of multiple deprivation.

**Table 3:** Multilevel model analyses of the outcome measures.

	Crude Model ^a^			Adjusted Model ^b^		
Outcome Measure	β or OR	95% CI	*p*	β or OR	95% CI	*p*
School day ST	**10.1 ^c^**	**−17.8 to −2.4**	**0.01**	**−9.0 ^c^**	**−17.7 to −0.2**	**0.04**
School day LPA	4.2 ^c^	−1.1 to 9.4	0.1	3.5 ^c^	−1.9 to 8.9	0.2
School day total PA	7.1 ^c^	−1.1 to 15.2	0.1	5.4 ^c^	−2.0 to 12.8	0.2
School day MVPA	1.9 ^c^	1.8 to 2.1	0.5	1.5 ^c^	−4.0 to 7.0	0.6
30 min MVPA/school day	**2.73 ^d^**	**0.36 to 2.20**	**0.03**	2.79 ^d^	0.49 to 2.71	0.07
Whole day ST	−0.2 ^c^	−23.4 to 22.9	1.0	−2.7 ^c^	−25.1 to 19.7	0.8
Whole weekday LPA	−2.7 ^c^	−14.2 to 8.8	0.9	−8.8 ^c^	−20.3 to 2.7	0.1
Whole weekday total PA	−2.5 ^c^	−19.7 to 14.7	0.8	−12.3 ^c^	−30.2 to 5.7	0.2
Whole weekday MVPA	−0.9 ^c^	−10.5 to 8.7	0.7	−4.1 ^c^	−13.9 to 5.7	0.4
CRF	**4.9 ^c^**	**0.8 to 8.9**	**0.02**	3.7 ^c^	−0.1 to 7.6	0.06
BMI z-score	0.0 ^c^	−0.2 to 0.2	0.8	0.0 ^c^	−0.2 to 0.2	1.0

Values reflect the intervention effects (i.e., between group differences) between baseline and post intervention. Values in bold denote beta (95% CI) and significance values of outcomes with significant intervention effects (*p* < 0.05). ^a^ Adjusted for group and baseline value of the outcome measure. ^b^ Additionally adjusted for confounding covariates. ^c^ β value. ^d^ OR. OR, odds ratio; CI, confidence interval; ST, sedentary time; LPA, light physical activity; MVPA, moderate to vigorous physical activity; CRF, cardiorespiratory fitness, BMI, body mass index.
